# Highlights from the Ninth European Breast Cancer Conference, Glasgow, 19–21 March 2014

**DOI:** 10.3332/ecancer.2014.426

**Published:** 2014-05-06

**Authors:** Elisabetta Munzone

**Affiliations:** European Institute of Oncology, Milan 20141, Italy

**Keywords:** breast cancer, epidemiology, screening, genetic testing

## Abstract

The Ninth European Breast Cancer Conference (EBCC-9), one of the largest breast cancer conferences in the world, was held in Glasgow in March 2014, and brought together the voices of doctors, researchers, nurses, and patients. All the major breast cancer advocacy groups and institutions were united in one forum (Europa Donna, the EORTC Breast Cancer group, and EUSOMA).

The Scientific Programme for EBCC-9 highlighted a holistic picture of breast cancer, including research, prevention, treatment, advocacy, and care. Participants were able to discover the most up-to-date developments and findings within the field for implementation into daily practice. Improvements in treatment, as well as enhanced access to care, underlie the sustained decreases in breast cancer mortality seen in 30 European countries from 1989 to 2010.

## Epidemiology of breast cancer

The GLOBOCAN project estimates that for 2012 there were 1.67 million cases of breast cancer worldwide. In Europe, including non-European Union countries, the latest estimates indicate that in 2012 there were 464,000 new cases of breast cancer, accounting for 28.8% of all cancer cases diagnosed in women and 13.5% of all cancer cases diagnosed overall. Globally, the latest estimates indicate that 7.5% of all cancer deaths in Europe in 2012 were attributable to breast cancer. EUROCARE-5 reported that the average five-year survival of European women diagnosed with breast cancer was 82% [1].

Prof Philippe Autier, from the International Prevention Research Institute, Lyon, France, explained that, although the decrease in deaths from breast cancer in 2010 was greatest in those countries with the highest mortality rates in the late 1980s, there were notable exceptions. In 1987–1989 breast cancer mortality rates were highest in England and Wales at 41.9 per 100,000, and lowest, at 20 per 100,000, in Romania. In 2008–2010 these rates were 25.4 and 22, respectively, indicating that mortality decreased by 40.8% in England, while it increased by 11.4% in Romania.

According to Prof Autier, screening has played an important role in decreasing the average size of tumours at detection. However, trends in the incidence of advanced breast cancer have remained stable, suggesting that screening does not succeed in detecting potentially life-threatening cancers at an earlier stage, and the number of breast cancers that have already metastasised in distant organs when first diagnosed has not decreased. Hence, these reductions in size simply represent the increasing incidence of small, early, non-life-threatening cancers that are detected by screening, and which give an overall impression that things are getting better in terms of outcomes. Therefore, decreased numbers of breast cancer deaths are largely due to improved treatments, not to screening.

Reductions in mortality from breast cancer were greatest in women of less than 50 years of age, with overall reductions of –71.3% to –21.4%, and smallest in women aged 70 and over (from –29.5 to +81.5), with breast cancer mortality among older women continuing to increase in many countries, particularly those in central and eastern Europe.

Yet in countries where major screening programmes were implemented after 2000 (e.g., Norway, Belgium, Switzerland, and Austria) considerable reductions in deaths from breast cancer were reported.

Nevertheless, the global burden of breast cancer remains immense in 2013, with over 1.6 million new cases being diagnosed annually. This burden has been increasing at a rate of 3.1% per year, and while the majority of new cases are diagnosed among women in developed countries, the 450,000 deaths per year from the disease are now equally divided between the developing and developed world.

Prof Peter Boyle, Director of the University of Strathclyde Institute of Global Public Health at the International Prevention Research Institute (iPRI) in Lyon, France, informed the conference that in Scotland the death rate from breast cancer is now at its lowest level in over 100 years, thanks to the contributions from a variety of sources, including the development and availability of effective treatments, increased awareness among women, the national NHS Breast Screening programme, and free access for all women to high-quality diagnostic and treatment facilities.

However, women in low income countries, and particularly in Africa, tend to seek medical attention for their disease only once it is at an advanced stage and has spread to other parts of the body, and by this stage the only option is palliation, which is often itself not available or, at best, not optimal. The World Breast Cancer Report 2012 collected data about groups of breast cancer patients from institutes throughout the world to develop a clearer picture of the current situation. In high-income countries, such as the United Kingdom and Australia, there were very few women diagnosed initially with stage III or IV disease, whereas in countries such as Kenya and Uganda almost all women with breast cancer presented at these later stages.

An increasing population size, longer life expectancy, a decrease in the stigma attached to a diagnosis of breast cancer, increases in awareness, and the introduction of early detection programmes in lower resource countries will inevitably lead to an increase in the numbers of new cases being diagnosed. Prof Boyle concluded by saying that epidemiology has a vital role to play both in elucidating the current situation and in bringing it to the notice of those who are able to do something about it.

## Importance of lifestyle choices

Recent studies showed that lifestyle choices are becoming more and more important in the prevention of breast cancer, avoiding a recurrence of breast cancer and helping to improve life as survivors of breast cancer.

According to the research presented to the Ninth European Breast Cancer Conference (EBCC-9) on 20 March, practising sport for more than an hour a day can reduce the risk of contracting breast cancer, and this applies to women of any age and any weight, and also from any geographical location. Compared with the least active women, those with the highest level of physical activity reduced their risk of breast cancer by 12%. Prof Mathieu Boniol, Research Director at the International Prevention Research Institute, Lyon, France, reported the results of a metaanalysis of 37 studies published between 1987 and 2013, representing over four million women. Although the results varied according to tumour type, the overall message was encouraging. However, in women taking hormone replacement therapy (HRT), the protective effect of exercise seemed to be cancelled out. Nevertheless, increased awareness of the side effects of HRT means that its use is decreasing in a number of countries, and this means that the beneficial effects of activity will most likely grow in the years to come. Physical activity is known to have a protective role in other cancers, as well as in disorders, such as cardiovascular disease. Although the mechanisms for its effect are unclear, the results are largely independent of body mass index (BMI), so the effect must be due to more than weight control. The researchers found no indication that breast cancer risk would decrease only when physical activity started at a young age. Both obesity and diabetes have adverse effects on outcomes in breast cancer patients who receive chemotherapy as primary treatment before surgery (neoadjuvant chemotherapy). Although a high BMI is known to have a negative impact on cancer development and prognosis, until now there has been uncertainty as to whether having a high BMI had an equal effect on patients with different types of breast tumours.

In this regard, Dr Caterina Fontanella, a trainee in medical oncology from the University of Udine (Italy) and a research fellow with the German Breast Group presented an analysis based on nearly 11,000 patients with early breast cancer treated with neoadjuvant chemotherapy. She showed that a high BMI adversely affects the chances of surviving without the breast cancer recurring or spreading to other parts of the body, although this detriment was not seen in those women had been diagnosed with HER2-positive disease. The researchers studied data from 8872 early breast cancer patients from the German Breast Group, and 1855 from a joint EORTC/BIG trial. All had received a modern treatment consisting of an anthracycline/taxane-based neoadjuvant chemotherapy, anti-HER-2 drugs, or hormone therapy according to the tumour type and national guidelines. The vast majority of the patients in this study received chemotherapy doses capped at a body surface area of 2.0 m^2^, which is often the limit when calculating doses. Final analysis of outcomes from the two groups in the joint study showed a significant decrease in distant disease-free survival (DDFS) or in distant relapse-free survival in patients with increased BMI in all tumour types, apart from those with HER2-positive tumours. According to Dr Fontanella the exception in this group can probably be explained by the impressive impact of anti-HER2 treatment. In a second study, Dr Fontanella and colleagues investigated the incidence of Type 2 diabetes in patients with early breast cancer at the time of diagnosis, as well as its effect on the outcome after neoadjuvant chemotherapy. Diabetes has been reported in 15% to 20% of elderly breast cancer patients, although in the group of just over 4000 patients studied it was considerably lower. Researchers found that patients with diabetes were more likely to have their cancer diagnosed at a more advanced stage, and this suggests that diabetes may affect the size of the tumour. Patients with diabetes were also found to have worse DDFS rates. Researchers hypothesise that hyperinsulinemia may encourage the growth of tumour cells by providing them with large amounts of glucose. Therefore, a strict control of blood sugar levels might be is essential to the successful treatment of breast cancer.

## The relevance of screening programmes

Analysis of data from the UK NHS Breast Screening Programme has shown significant variations in the outcomes of treatment for women with ductal carcinoma *in situ* (DCIS) between UK hospitals.

Dr Jeremy Thomas, a consultant pathologist at the Western General Hospital, Edinburgh, UK, reported that although the majority of women with DCIS received the correct surgery for their disease, large numbers of women were undergoing mastectomy for DCIS either as a result of failed breast conservation surgery or for tumours that turned out to be smaller than 20 mm in diameter, and therefore should normally have had a lumpectomy rather than a mastectomy. Usually, decisions about how women with DCIS should be treated are taken in multidisciplinary teams that include radiologists, pathologists, surgeons, oncologists, and nurses. Nevertheless, Dr Thomas reported that, in some hospitals, the discussions in the multidisciplinary teams are not looking in enough detail at the results from the mammograms and pathology to make the right decision about the best surgical treatment for these women. Dr Thomas and his colleagues in The Sloane Project—a multidisciplinary, UK-wide prospective audit of screen-detected non-invasive breast cancers and atypical hyperplasias—collected data from 8313 patients with DCIS detected during screening from 2003 onwards.

They reported that of 6633 women who were treated with breast conservation surgery, 799 (12%) required a subsequent mastectomy. Failed breast conservation surgery accounted for one-third of all the women who ended up having a mastectomy, and was usually because of under-estimation of the extent of the disease from the mammograms. The total number of women who had mastectomies, including those for whom mastectomy was the first course of action as well as those who had failed breast conservation, was 2479; of these, 510 (21%) had mastectomies for tumours smaller than 20 mm in diameter, which would normally have been better treated with a lumpectomy. To analyse the variations between hospitals, Dr Thomas and his colleagues selected 57 hospitals that had submitted data on the highest number of patients. These hospitals had data on between 50 and 387 cases each, making a total of over 6000 patients, which was around 80% of the total number of women being studied. The proportion of failed breast conservation surgery in these hospitals ranged from 3% to 32%, while the proportion of mastectomies for small tumours ranged from 0 to 60%. The researchers then divided the 57 hospitals into three subgroups, with 19 hospitals in each, based on how often the wrong surgery was carried out. In the high frequency group for failed conservation surgery there was an adverse outcome on average in 22.3% of cases, in the medium group in 13.4% of cases, and in the low group in 7% of cases. Dr Thomas concluded by affirming that the UK NHS Screening Programme is working well and that the right surgical decisions are being made in the majority of cases. However, the significant variation between hospitals shows that the approach can be improved.

A study of over 50,000 women participating in the UK NHS Breast Screening Programme has found that, while three-yearly screening intervals are appropriate for the majority of women, approximately one-third of women are at higher risk of developing cancer and might benefit from more frequent mammograms. Prof Gareth Evans, from the University of Manchester (UK), reported that identifying the degree of risk of developing breast cancer in individual women would enable healthcare professionals to target screening and preventive measures better. To see if this was feasible, he and his colleagues from Manchester and Queen Mary University of London (UK) collected extra information from women attending routine breast screening in Manchester and who had agreed to participate in the Predicting Risk Of breast Cancer At Screening study. A questionnaire was used to collect information on important breast cancer risk factors, such as family history and lifestyle; where appropriate, genetic information was collected by analysing saliva samples; breast density was measured from the mammogram and given a ‘visual assessment score’ (VAS), which indicated the percentage of dense tissue in the breast. The study started in 2009 and the first 53,467 women were included in the analysis reported at EBCC-9. During this time 634 women developed breast cancer. The NHS Breast Screening Programme is usually open to women between the ages of 47 and 73 years, and the ‘normal’ risk of developing breast cancer within the next ten years varies from 2.4% at the age of 47years to 3.5% at the age of 70 years. The risk factor questionnaire indicated that 676 (1.4%) women had a high risk of developing breast cancer of 8% or more over the next ten years, with a further 4591 (8.6%) women having a moderately increased risk of between 5% and 8%.

When Prof Evans and his colleagues combined the results from the risk factor questionnaire and VAS to make the results more accurate at defining the women’s risks, they found that 1280 (2%) of women had a high risk (8% or more) of developing breast cancer with 29 (2.3%) developing breast cancer. There were 14,720 women with an above average risk (over 3.5%) of developing breast cancer in the next ten years and, indeed, 267 (1.8%) had developed breast cancer in the four-year period from 2009. This left 36,748 women with average or below average risk of developing breast cancer, and, of these, only 371 (1%) developed breast cancer. When looking specifically at the 3432 women in this group who had a low (less than 1%) ten-year risk, only 10 (0.3%) developed breast cancer during the four years. The researchers also found that among the 36,748 women at average or below average risk, only 45 cancers that had started to spread to the lymph nodes were found during the four years, which was equivalent to about three per 100,000 women a year, compared with around 11 per 100,000 women a year in those at above average risk. Proportions of cancers that were further advanced were higher in the above average risk women: 32% compared with only 19% in women at average or below average risk. Therefore, these results suggest that three-yearly screening is very effective for around 70% of the female population, but that those women who have a higher than average risk of developing breast cancer probably require more frequent screening, particularly as more advanced cancers were detected in these women. Screening should be annual for the small proportion of women who have an 8% or greater risk of developing cancer over the next ten years.

Dr Gerrit-Jan Liefers, a surgical oncologist and head of the geriatric oncology research group at Leiden University Medical Centre reported on the screening programme in the Netherlands. The Netherlands Breast Cancer Screening Programme was extended in 1998 to include women up to the age of 75 years. Dr Liefers and his colleagues looked at results from The Netherlands Cancer Registry for 25,414 women aged between 70 and 75 years, who were diagnosed with breast cancer between 1995 and 2011. They found that after the extension of the upper age limit, the incidence of early stage breast cancer (stages 0, I, and II) increased significantly from 260 cases per 100,000 women in 1995 to up to 382 cases per 100,000 women in 2011. Meanwhile, the number of advanced stage breast cancers (stages III and IV) did not change significantly: in 1995 there were 59 cases detected per 100,000 women compared to 53 per 100,000 in 2011.

## Importance of genetic testing

Genetic analyses of results from 1125 postmenopausal women being treated for oestrogen responsive breast cancer have shown that some of them are more likely than others to have a late recurrence of their cancer and might benefit from ten years of hormone therapy rather than five. Prof Mitch Dowsett reported his research showing that women with HER2-negative and ER-positive breast cancer had more than double the risk of their cancer recurring between five and ten years after surgery and five years of adjuvant hormone therapy. Data suggest that these patients, who are those that appear to benefit most from the current standard five years of endocrine treatment, may also benefit from adjuvant hormone treatment that extends beyond five years. The work is a collaboration between his team and that of Prof Jack Cuzick at the Wolfson Institute of Preventive Medicine, Queen Mary University of London, UK. The findings are the latest to come from the ATAC trial (Arimidex, Tamoxifen Alone or in Combination), a double-blinded phase III clinical trial that randomly assigned postmenopausal women with early, oestrogen receptor positive (ER+) breast cancer to receive the hormone therapies anastrozole or tamoxifen, or a combination of the two [2]. Prof Dowsett and his colleagues at The Royal Marsden, in London used data from the OncotypeDx 21-gene Recurrence Score to analyse the genetic make-up and to predict the likelihood of cancer recurring within ten years in these women. OncotypeDX has been used for over 350,000 tests and the ATAC team had previously shown that its prediction of recurrence was poorer in the second five years after a patient’s diagnosis than in the first five years. The researchers wanted to find out the reason for this, and to do so they determined the relationship between the expression of the individual genes and gene modules and early (up to five years) and late (between five and ten years) recurrence rates in women with ER+ HER2– breast cancer. They assessed the gene expression and recurrence rates in 1125 women in the ATAC trial, who had an average of ten years of follow-up. Nearly 90% of the women were HER2-negative and there were 215 recurrences during the ten years. They found that recurrence rates were highest in the first five years for women with HER2+ breast cancer, compared with the subsequent five years, but for women with HER2– cancer, the recurrence rates were higher between five and ten years. Prof Dowsett reported that among women with tumours most sensitive to oestrogen, with a high E-module score, the recurrence rate more than doubled from 5.7% in the first five years to 13.6% in the subsequent five years. However, if they had a low E-module score, there was little difference in recurrence rates between the first five years and the next five years: 10.3% versus 12.3%. The researchers highlighted that, despite similar overall recurrence rates for patients with ER+ tumours between the first five years and the next five to ten years, there were important differences between groups of tumours with different genetic expression profiles. Importantly, HER2– tumours that are very sensitive to oestrogen are usually considered to be relatively low risk, yet these were the tumours that showed an increase in recurrence after five years, which coincided with the cessation of adjuvant hormonal therapy. It is commonly thought that the reduction in recurrence achieved by five years of endocrine therapy ‘carries-over’ into the next five years. These results suggest this effect may differ markedly between different groups of ER+ tumours. Better predictors of recurrence than the OncotypeDX and others currently being used should be possible based on the different recurrence rates of different groups of tumours and their different sensitivity to endocrine therapy. These findings could change clinical practice: women with HER2–, high oestrogen signalling breast cancer might be considered for adjuvant hormone therapy that is extended to ten years. However, the results need to be confirmed in other sets of tumours first particularly in those patients that are participating in ongoing trials of extended versus no extended adjuvant therapy.

## Conclusion

The EBCC-9—the largest breast cancer conference outside the United States—was made unique by including patient advocates together with breast cancer professionals. The aim of the conference was to disseminate information directly to patient advocates and enable health-care professionals to implement new findings in daily practice.

The conference provided a unique multidisciplinary setting for all participants to discuss, inform, and educate themselves about this evolving disease landscape. Moreover, the conference provided the opportunity to implement new findings into daily practice, making a tangible difference for the individual patient.

The next conference (EBCC-10) will be held in Amsterdam, The Netherlands, 9–11 March 2016.
